# Sudden death due to ventricular double rupture as a complication of inferior acute myocardial infarction: A case report

**DOI:** 10.1097/MD.0000000000005757

**Published:** 2016-12-30

**Authors:** Shi-Jian Chen, Chen Zhang, Qing-Tao Meng, Yong Peng, Mao Chen

**Affiliations:** Department of Cardiology, West China Hospital, Sichuan University, Chengdu, China.

**Keywords:** acute myocardial infarction, sudden death, ventricular double rupture

## Abstract

**Rationale:**

Ventricular double rupture (VDR) is a rare but lethal mechanical complication of acute myocardial infarction (AMI). The early identification and timely treatment of VDR remain challenging problems. We present a case of AMI with VDR and briefly review the characteristics and prognosis of this life-threatening disease.

**Patient concerns and Diagnoses:**

A 77-year-old male presented to our hospital with a 4-day history of severe dizziness, mild chest tightness, and dyspnea. An inferior AMI was diagnosed.

**Interventions and Outcomes:**

On the second hospital day, hypotension and a new cardiac murmur was found. The emergency echocardiographic study disclosed a ventricular septal defect. Soon after that the patient suddenly died of ventricular free-wall rupture.

**Lessons:**

In patients with AMI complicated by a septal perforation in the apical region, close to the septum-free wall junction, special attention should be paid to the great risk of VDR. Other high risk factors included advanced age, delayed reperfusion, and inferior infarction. Sufficient evaluation of the risk factors, close monitoring of vital signs, early identification of the specific symptoms, and timely treatment are the key points for the effective prediction and prevention of VDR.

## 1 Introduction

Ventricular rupture (VR), a rare but fatal mechanical complication of acute myocardial infarction (AMI), is the second leading cause of in-hospital death and is responsible for as many as 15% of total early deaths in patients with AMI.^[1,2]^ The types of VR include left ventricular free wall rupture (FWR), ventricular septal rupture (VSR), and papillary muscle rupture. In addition, ventricular double rupture (VDR) is defined as the coexistence of 2 of the above-mentioned rupture types.

We present the case of a male patient who suffered sudden death due to the coexistence of VSR and FWR complicating inferior AMI. The point of this case is to highlight that VSR in the apical region, which is close to the septum–free wall junction, is a precursor of VDR, and that an inferior infarction is an important poor prognostic factor for VDR.

## 2 Case presentation

**Figure 1:**
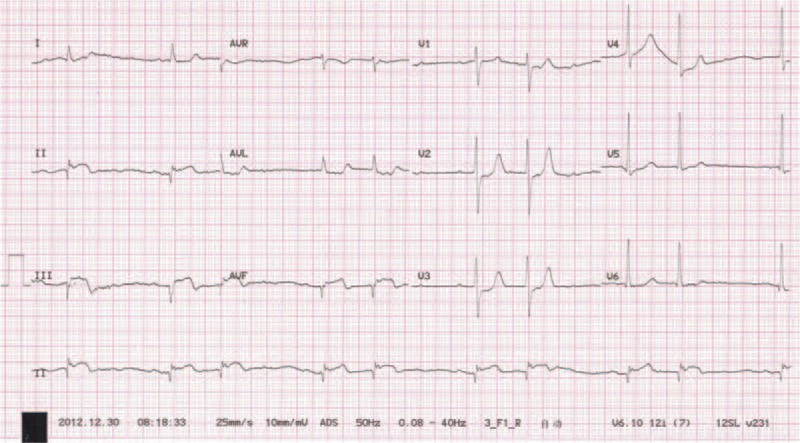
Electrocardiogram showing acute myocardial infarction, with ST-segment elevation in the inferior leads, and second-degree atrioventricular block.

**Figure 2:**
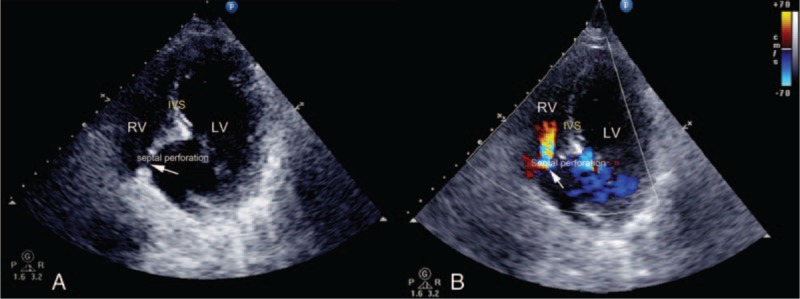
(A) Nonstandard left ventricular short-axis section at the apical level showing a ventricular septal defect (arrow). The site of septal perforation was the apex, adjacent to the septum–free wall junction. (B) Color Doppler shows a left-to-right shunt at the level of the ventricular septal perforation.

**Figure 3:**
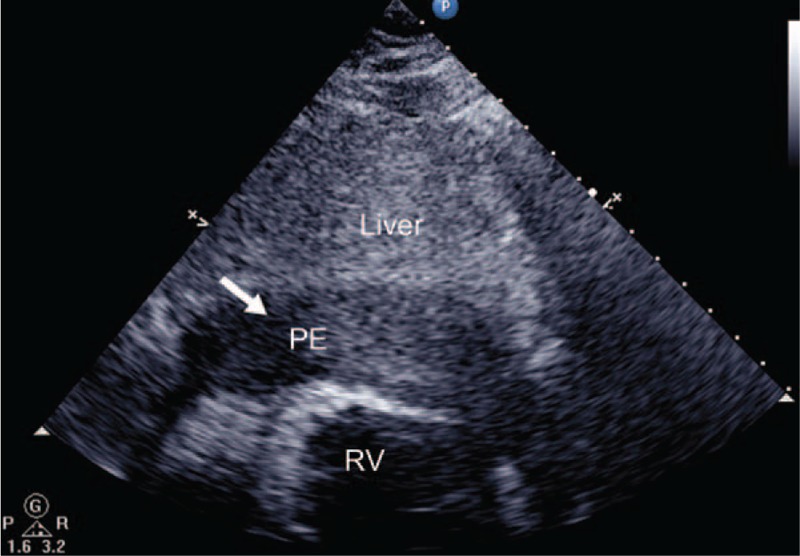
Echocardiography showing a massive hemorrhagic pericardial effusion (arrow).

A 77-year-old male with diabetes, hypertension, ischemic stroke, chronic obstructive pulmonary disease, and chronic kidney disease presented to our hospital with a 4-day history of severe dizziness, mild chest tightness, and dyspnea. Clinical examination showed an obesity body habitus and a respiratory rate of 22 breaths/min. His heart rate was 82 beats/min, and his blood pressure was 135/68 mm Hg. Chest expansion and breath sounds were mildly reduced bilaterally, with some wet rales in the lower region. Auscultation revealed a normal heart sound with no murmur. A smooth liver edge was palpable below the right costal margin and there was mild bilateral leg edema in the ankles. The electrocardiogram (ECG) revealed convex upwards ST-segment elevation with T-wave inversions and pathological Q waves in leads II, III, and aVF, and second-degree atrioventricular block (Fig. 1). Additionally, cardiac troponin T and pro–brain natriuretic peptide levels were clearly increased. An inferior AMI was diagnosed. Because of the delayed admission (4 days after symptom onset) and the stable clinical conditions, the patient was treated conservatively. On the second hospital day, hypotension occurred in the morning and cardiac auscultation indicated the onset of a new pansystolic murmur at the inner proximal apex. An emergency bedside echocardiographic study disclosed a ventricular septal defect with the site of septal perforation at the apex, adjacent to the septum–free wall junction (Fig. 2A and B). Emergency surgery was considered, but the patient suddenly lost consciousness and palpable pulse approximately 50 minutes later. Cardiopulmonary resuscitation was performed immediately, and electromechanical dissociation was observed on the ECG monitor. Bedside echocardiography displayed a massive hemorrhagic pericardial effusion (Fig. 3) and the near disappearance of cardiac systolic movement. Pericardiocentesis was performed, but there was no recovery of spontaneous circulation. The patient died of VDR.

## 3 Discussion

Owing to the increasing use of reperfusion therapy, the rational administration of medicines, and the improvements in surgical techniques, VR tends to occur with decreasing frequency.^[1,3]^ However, as its mortality is reported to remain almost unchanged in the past few decades, VR is still recognized as a devastating complication of AMI.^[4–6]^

VDR, with most frequent combination with FWR and VSR, is observed in approximately 3% of patients with FWR, 16% of those with VSR, and 0.3% of all patients with AMI.^[7]^ FWR, the most common type of VR with an estimated incidence being approximately 2% to 10%, causes 3% to 20% of overall mortality and 15% of the in-hospital mortality after AMI.^[1,8]^ The incidence of VSR decreased from 1% to 3% in the prethrombolytic era to 0.17% to 0.31% in the era of reperfusion therapy.^[4–6,9–11]^ Previous research studies of autopsied hearts demonstrated that VSR was accompanied by FWR in 17% of cases.^[12]^ The outcomes of VSR are extremely poor in both medically managed patients and those treated with surgery.^[4,13]^ Patients with VSR have significantly higher mortality at 30 days and 1 year, and are more likely to suffer from shock and congestive heart failure.^[5]^

Several risk factors for VR in post-AMI patients were found in previous studies, including advanced age, female sex, prior stroke, a history of hypertension and chronic kidney disease, increased heart rate, worse Killip class at admission, persistent ST-segment elevation, anterior or transmural infarction, total occlusion of the infarct-related artery, continued physical activity postinfarction, and delayed treatment.^[2,3,7]^ The role of thrombolytic agents and β-blockers in the development of VR is controversial.^[3,7]^ Patients with VR are less likely to have a history of diabetes, prior myocardial infarction, or heart failure.^[14,15]^

The mean time interval between AMI and FWR appears to be 3.7 ± 4.4 days, while that between AMI and VSR is 4.1 ± 3.6 days.^[1,16]^ In fact, in half of all cases, VR may occur as early as a few hours after infarction.^[8]^ Thrombolytic therapy may shorten the time interval and accelerate the onset of VR via myocardial hemorrhage.^[2]^ Moreover, advanced age, inferior AMI, cardiogenic shock, cardiac arrest, right ventricular dysfunction, and a long interval between onset and surgery are considered to be poor prognostic factors after VR has developed.^[5,17,18]^

The patient in the present report unfortunately had numerous risk factors for post-AMI VR: advanced age, complex clinical conditions, delayed admission, no ST-segment resolution, and no timely reperfusion or surgery. Most notable among these risk factors may have been the site of septal perforation, which lay in the apical region, close to the septum–free wall junction. Tanaka et al found that the perforation site of VSR played an important role in the development of FWR in 4 patients; the authors advised that septal perforation at the junction between the ventricular septum and the free wall is a precursor of VDR that mandates surgical treatment as early as possible.^[7]^ In addition, our case presented sudden death due to VDR as a complication of inferior ST-segment elevation AMI. VSR occurs in the setting of inferior infarction in approximately 30% of all cases.^[5]^ Since an inferior infarction is more likely to be associated with right ventricular dysfunction and complex VSR,^[12]^ patients with an inferior infarction tend to have worse outcomes.

## 4 Conclusion

For patients with AMI complicated by a septal perforation in the apical region, close to the septum–free wall junction, special attention should be paid to the great risk of VDR. The outcomes of VDR remain extremely poor, even in patients who undergo surgical repair. Sufficient evaluation of the risk factors, close monitoring of vital signs, early identification of the specific symptoms, and timely treatment are the key points for the effective prediction and prevention of VDR.
